# Single cell imaging-based chromatin biomarkers for tumor progression

**DOI:** 10.1038/s41598-021-02441-6

**Published:** 2021-11-29

**Authors:** Saradha Venkatachalapathy, Doorgesh S. Jokhun, Madhavi Andhari, G. V. Shivashankar

**Affiliations:** 1 Mechanobiology Institute and Department of Biological Sciences, National University of Singapore, Singapore, 117411 Singapore; 2 Department of Biological Sciences, Indian Institute of Science Education and Research Kolkata, Mohanpur, West Bengal 741246 India; 3 FIRC Institute for Molecular Oncology, 20139 Milan, Italy; 4 Department of Health Sciences and Technology, ETH Zurich, Zurich, Switzerland; 5 Paul Scherrer Institut, 5232 Villigen, Switzerland

**Keywords:** Biophysics, Cancer, Computational biology and bioinformatics, Biomarkers

## Abstract

Tumour progression within the tissue microenvironment is accompanied by complex biomechanical alterations of the extracellular environment. While histopathology images provide robust biochemical markers for tumor progression in clinical settings, a quantitative single cell score using nuclear morphology and chromatin organization integrated with the long range mechanical coupling within the tumor microenvironment is missing. We propose that the spatial chromatin organization in individual nuclei characterises the cell state and their alterations during tumor progression. In this paper, we first built an image analysis pipeline and implemented it to classify nuclei from patient derived breast tissue biopsies of various cancer stages based on their nuclear and chromatin features. Replacing H&E with DNA binding dyes such as Hoescht stained tissue biopsies, we improved the classification accuracy. Using the nuclear morphology and chromatin organization features, we constructed a pseudo-time model to identify the chromatin state changes that occur during tumour progression. This enabled us to build a single-cell mechano-genomic score that characterises the cell state during tumor progression from a normal to a metastatic state. To gain further insights into the alterations in the local tissue microenvironments, we also used the nuclear orientations to identify spatial neighbourhoods that have been posited to drive tumor progression. Collectively, we demonstrate that image-based single cell chromatin and nuclear features are important single cell biomarkers for phenotypic mapping of tumor progression.

## Introduction

The tumor microenvironment is a dynamic landscape in which the mechano-chemical properties of the surrounding extracellular matrix evolve throughout cancer progression^1^. Such extracellular matrix remodelling alters the nuclear and chromatin organization to bring about differential gene expression programs that influence the cancer as well as the stromal cells^2^. The 3-dimensional packing of the genome regulates the transcriptional activity of the cell and thereby, the cell state and function^3,4^. DNA is folded in a hierarchical manner, starting with the metre-long molecule which is organized into topologically associated domains (TADs) which further form micron-sized higher order structures comprising of functionally distinct highly condensed heterochromatin and less condensed euchromatin regions^5,6^.This 3D chromatin structure is maintained and tuned by the cytoskeletal to nuclear links in partnership with the epigenetic machinery^7,8^. Hence in tissues, microenvironment changes as perceived by cell–matrix interactions and/or via biochemical signalling, result in precise re-arrangement of the 3D chromatin to elicit robust transcriptional responses to environmental stimuli^9^.

The tight coupling between the stromal microenvironment and the single cell chromatin organization is involved in the maintenance of tissue homeostasis and alterations to this balance has been shown to lead to onset and progression of diseases such as cancer. Defects in the structural links between the matrix, cytoskeleton and nucleus either due to mutations or altered nuclear-mechanotransduction results in changes to the chromatin packing beyond homeostatic bounds^10,11^. Such defects disrupt the normal cellular response to signals and promote pathological phenotypes as seen in many diseases such as laminopathies, muscle dystrophies and cancers^12,13^. Importantly, the metastatic potential of the cancer cells is directly linked to their mechanical properties such as cell–matrix adhesions and the nuclear mechanotransduction pathways^14^. Such alterations during tumour progression are correlated with irregularities in chromatin compaction and curvature of the nuclear boundary and are used as standard visual features by pathologists for diagnosis^15^. Importantly the tumor microenvironment elicits complex mechanical interactions between stromal cells and cancer cells leading to localized changes to the biophysical properties of the tissue such as stiffness^1,16^. Such changes to the normal tissue surrounding the tumor has been shown to be vital for tumor progression^17,18^. While histopathology images provide robust biochemical markers for tumor progression in clinical settings, a quantitative single cell score depicting spatial organization of chromatin inside individual nuclei and the long range mechanical coupling within the tumor microenvironment is missing.

In this paper, we first built an image analysis pipeline that performs nuclear segmentation and computes single cell features that describe nuclear morphology and chromatin organization. We show that tissue biopsies stained using Hoechst, a DNA binding dye, improves the classification accuracy. Using the nuclear morphology and chromatin organization features, we constructed a pseudo-time model to identify the chromatin state changes that occur during tumour progression. We also built a single-cell mechano-genomic score that characterises the cell state during tumor progression from a normal to a metastatic state. Using immunostaining, we show that the mechano-genomic score was different for cells expressing standard cancer biomarkers. Further, we used the nucleus as a sensor of local tissue mechanics to identify spatial neighbourhoods in intermediate stages of breast cancer that have been posited to drive tumor progression. Collectively, we show that the fluorescence-based DNA images provide quantitative single-cell features that can be used as robust and interpretable biophysical biomarkers in complex tumor microenvironments.

## Results

### Imaging based nuclear and chromatin features as single cell biomarkers in tissue biopsies

**Figure 1:**
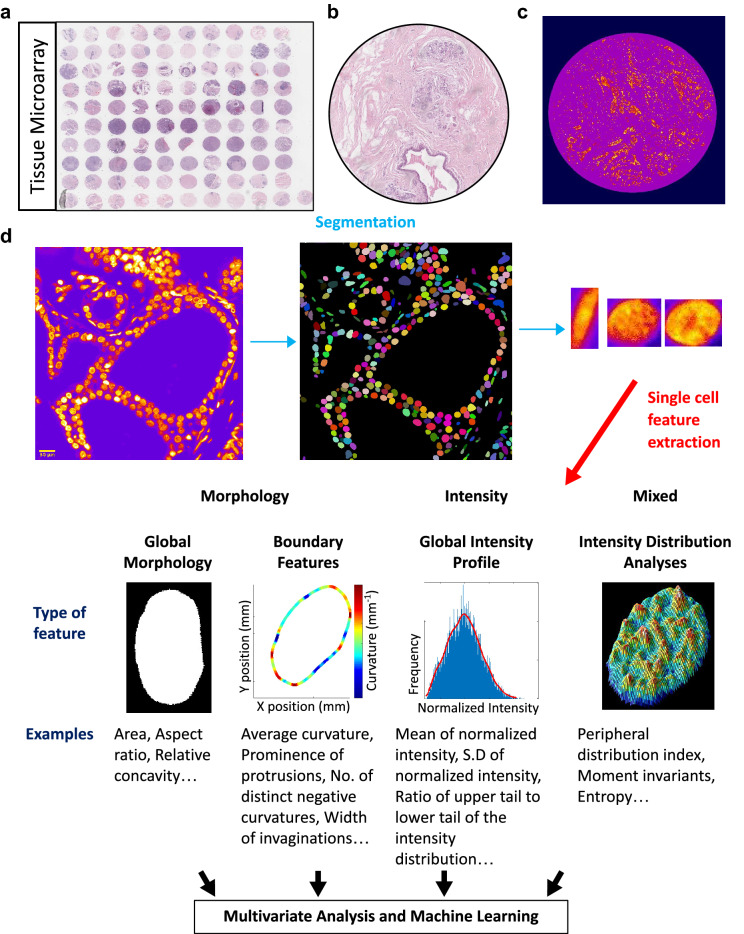
Imaging based nuclear and chromatin features as single cell biomarkers in tissue biopsies. (**a**) An overview of the biomax Tissue Microarray (TMA). (**b**) Representative H&E stained TMA image. (**c**) Representative Hoescht stained TMA image. (**d**) The first module of the pipeline automates the thresholding, segmentation and cropping of individual nuclei from large images. A library of individual nuclei is created and passed to the second module where genome architectural (morphological and textural) features are extracted from each nucleus. The consolidated single cell data is then used for further analysis.

Our working hypothesis is that the spatial organization of chromatin inside an individual nucleus contains sufficient information about the functional state of the cell and can therefore be exploited for developing image-based single cell diagnostic tools. A prerequisite for such platforms would be the ability to extract enough information at the scale of single nuclei (typical size: 10 µm) when provided with large tissue images (typical size of a biopsy: 1 mm). The data would then have to be processed and made suitable for training, optimizing and testing various machine learning models. Figure 1 schematically describes a pipeline developed for this purpose. The first module automates the segmentation of individual nuclei from large images. In this paper, we have used two approaches (intensity and model based) to perform instance segmentation of nuclei in a given image. The intensity based approach uses classical computer vision methods namely, thresholding watershed and pixel connectivity object detections. For more crowded and large images, we used the StarDist model based approach which implements a U-net architecture based convolutional neural network to identify convex polygons^19^. To train and test the network, we used images from the 2018 DSB nuclear segmentation challenge^20^, in addition to 1000 nuclei from 10 different TMAs for which the ground truth labels were manually generated in house. Our model has a segmentation sensitivity of 82% on the validation dataset (Fig. S1A). Following segmentation, a library of individual nuclei is created and passed to the second module where nuclear morphology and chromatin features are extracted from each nucleus. These features can be categorized in four groups, namely, global morphological features, nuclear boundary features, global intensity features and intensity distribution features. Examples of each category are provided in Fig. 1 and details about individual features can be found in Supplementary Table S1. Once the whole library is analysed, the consolidated single cell data is passed on to the third module for statistical analysis and machine learning.

The claim that genome organization, at the scale of the nucleus, holds enough information about cellular functions was supported by two tests. The first test was to see if these features can discriminate between three different normal human cell lines: one epithelial (HME) and two fibroblasts from different organs, HMF3A from the breast and BJ from the foreskin. Linear Discrimiant Analysis (LDA) using these features was able to classify the cell types with an accuracy of 60% for fibroblasts and 80% for the epithelial cells (Fig. S1B). This result is in line with the recent literature that showed that the chromatin organization is cell-type specific^21^. The second test was performed to check the sensitivity of these features. Here, a normal cell was exposed to the secretome of two different grades of cancer: fibrocystic and metastatic. After 3 days of exposure we could accurately distinguish the cells exposed to the metastatic secretome with 80% accuracy. (Fig. S1C). This is in line with recent literature that has highlighted the importance of the cross talk between cancer cells and stromal cells in the tissue microenvironment^22–24^. These results support our hypothesis that the genome organization, at the scale of the nucleus, is highly correlated with cell state.

### Hoechst staining improves the sensitivity to discriminate tumor progression

**Figure 2:**
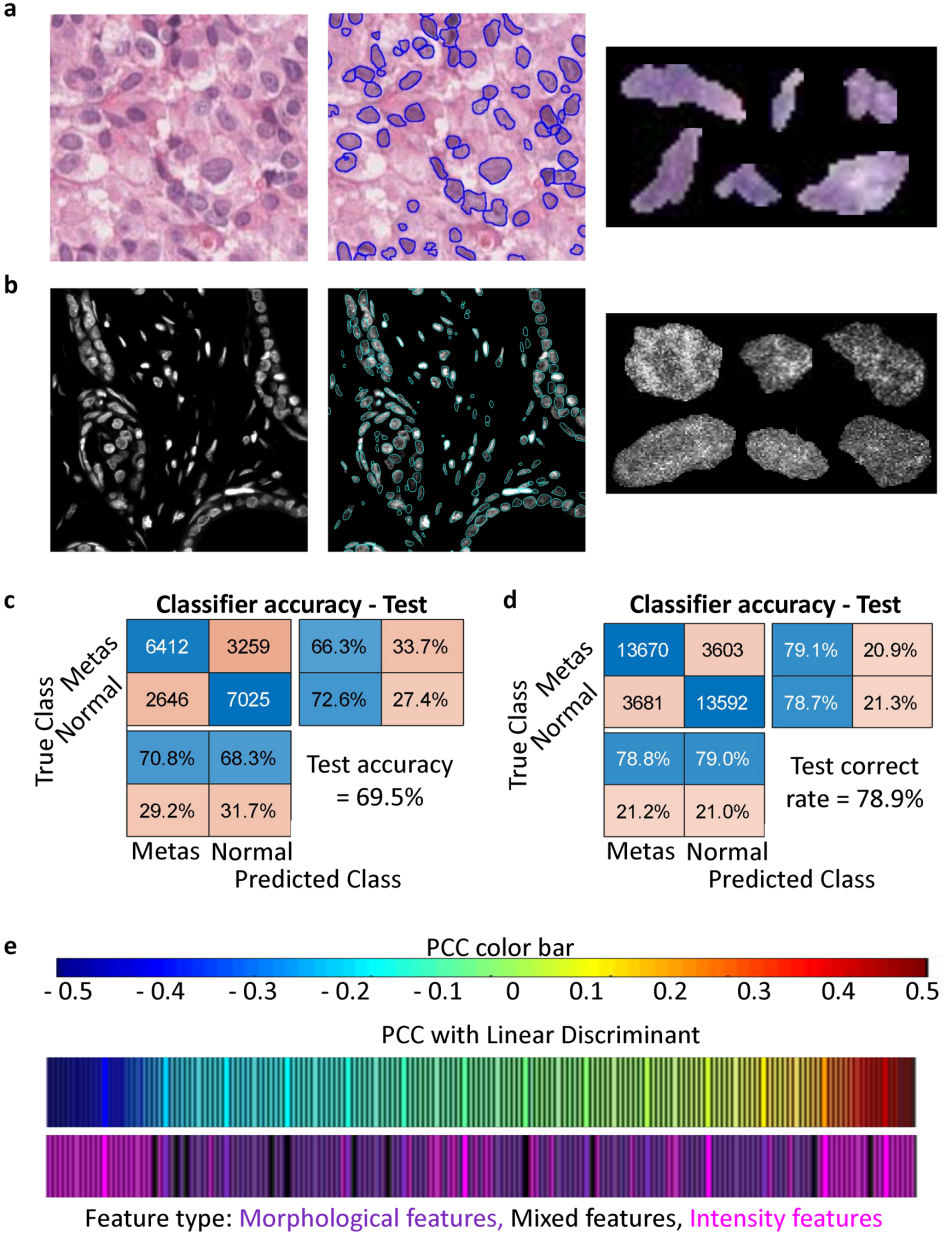
Hoescht staining improves the sensitivity to discriminate tumor progression. Representative crop of an H&E-stained (**a**) and Hoescht stained (**b**) tissue biopsy image (left). Marked in blue are the regions detected as nuclei for downstream segmentation and analysis. Representative crops of individual nuclei segmented are shown on the right side. Confusion matrices depicting the performances of the linear discriminant classifier in classifying nuclei from normal and metastatic tissues according to their nuclear features in H&E-stained tissues (**c**) and Hoescht stained tissues (**d**). (**e**) Correlation between nuclear features and the linear discriminant axis for the Hoescht-stained nuclei. The features have been colour-coded and grouped into morphological features, mixed features and intensity features.

After demonstrating that single cell nuclear features could discriminate cells in-vitro, we next tested if they could distinguish healthy and tumorigenic cells from patient derived H&E stained breast tissue biopsies (Fig. 2a). Individual nuclei were segmented using the intensity based approach from large images of metastatic as well as normal tissues and nuclear morphological and chromatin organization features were extracted. Principal Component Analysis (PCA) was performed on a training dataset consisting of 38,682 nuclei randomly selected from each of the two classes and the principal components were used to train a linear discriminant model, which was subsequently evaluated on a test dataset of 9671 previously unused nuclei from each class. The model was found to have similar classification accuracies (~ 70%) on both the training and the test sets (Fig. 2c and S1a). Further, intensity/texture based features were found to be the most correlated (positive and negative) with the linear discriminant values (Fig. S2c).

Though H&E staining is more commonly used in clinical settings, we hypothesized that stains whose intra-nuclear intensities are more sensitive to local chromatin compaction might reveal more information about the state of the cell. This is in line with our conclusion from the previous section as well as the large number of studies which have linked changes in chromatin organization with disease states^25–28^. Unstained TMA with slices from the same biopsy cores as the previously used H&E stained TMA were stained with Hoescht, a DNA-binding dye routinely used to study intra-nuclear chromatin organization (Fig. 2b). The samples were then imaged, the images segmented and the individual nuclei analysed by our pipeline. Chromatin architectural features of 69,092 nuclei randomly selected from normal and metastatic tissues were pooled for the training dataset and PCA-LDA was performed. The accuracy of the classifier was found to be around 79% for both the training and the test nuclei (Figs. 2d, S2b). Further, similar to the LDA trained on H&E images, intensity features were found to be the most correlated (positive and negative) with the linear discriminant values of the Hoescht-stained nuclei (Fig. 2e). Taken together, our results demonstrate that nuclear features can discriminate nuclei from normal and metastatic tissues. Importantly, substituting H&E staining with Hoescht resulted in ~ 10% gain in classification accuracy. Henceforth, we use only Hoechst stained TMA slices.

### Building a trajectory of chromatin reorganization associated with breast cancer progression

**Figure 3:**
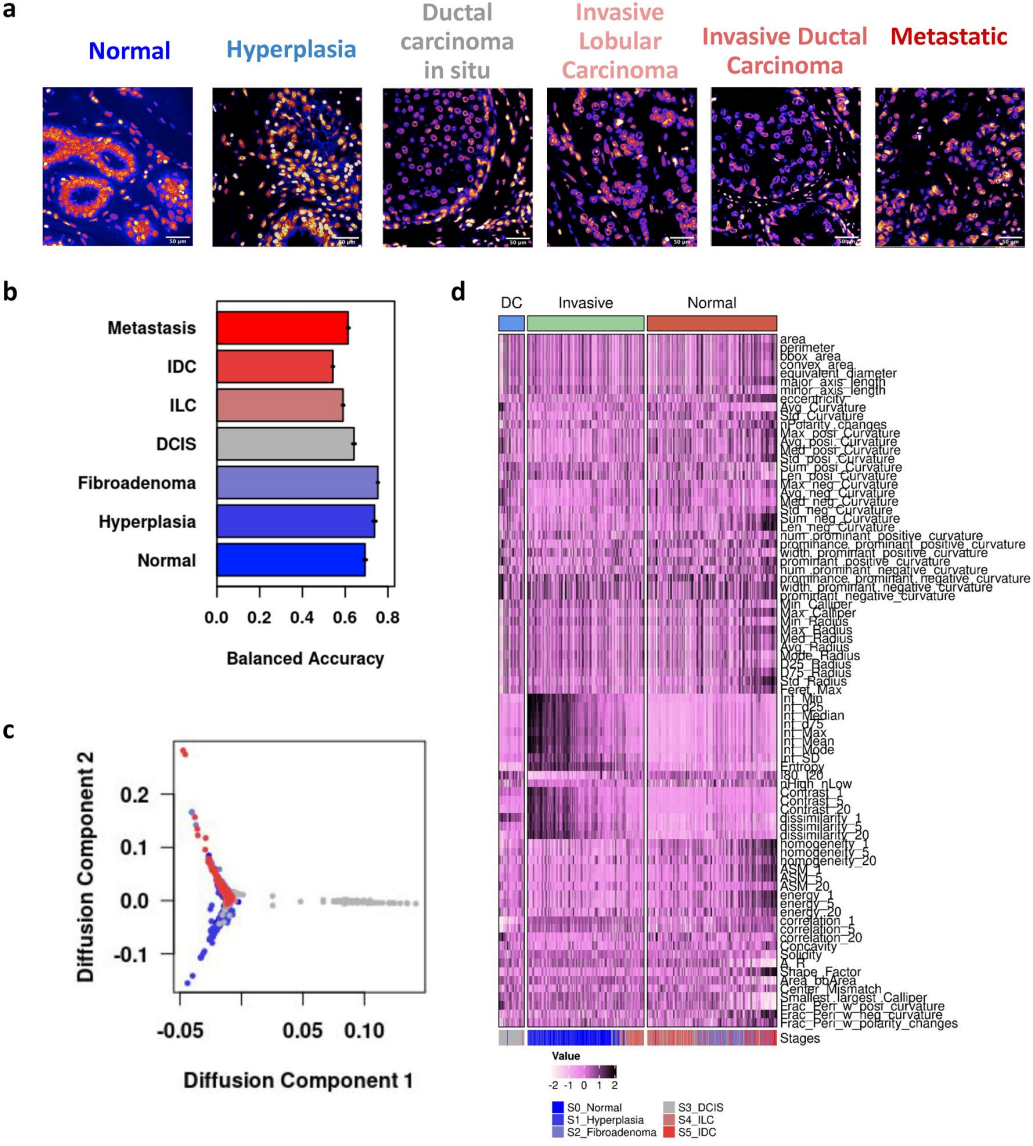
Building a trajectory of chromatin reorganization associated with breast cancer progression. (**a**) Representative crops of Hoescht -stained tissue biopsy images of different stages of breast cancer. (**b**) Prediction accuracy of the linear discriminant classifier on the test dataset. Error bars denote the standard error of classification in across 5 cross validation tests. (**c**) Diffusion Map of nuclei from various stages of cancer. Each dot represents one cell and the color code is displayed as an inset. (**d**) Heatmap depicting the branch-wise changes to representative nuclear morphology and chromatin organization features. The color code is indicated near the bottom. Note that each column represents a single cell. The column-side color bar at the bottom of the heatmap represents the stage of cancer.

During cancer progression, while cancer cells undergo tumorigenic transformations within the tumor, the surrounding normal cells are also altered. Tumor cells activate fibroblasts in the stroma and also recruit macrophages and other immune cells^17,22^. Hence, cancerous tissues will have a distinct composition of cell types and cell states, even among non-cancer cells. We hypothesized that such population level shifts can be traced using nuclear morphology and chromatin organization features. Towards this end, nuclei were segmented from TMA stained with Hoescht (see “Materials and methods”). Nuclear morphology and chromatin organization features were computed for nuclei from biopsies clinically annotated as (i) Normal, (ii) Hyperplasia (iii) Fibroadenoma, (iv) Ductal Carcinoma In Situ (DCIS), (v) Invasive Lobular Carcinoma (ILC), (vi) Invasive Ductal Carcinoma (IDC) and (vii) Metastatic (Fig. 3a). Normal and Hyperplasia tissues are clinically healthy, and these contain only normal cells. However, it should also be noted that biopsies annotated as any of the disease stages are bound to have cells from the other classes as well.

We then obtained a consolidated dataset of multivariate features that describes the nuclear morphology and chromatin organization of each cell in the TMAs using our analysis pipeline. We then split this into training and test datasets (see “Materials and methods”). Using the training data, we trained three different classifiers: Linear discriminant analysis (LDA), Random forest (RF) Classifier and an Artificial Neural Network (ANN). The performance of the classifiers was evaluated by computing the area under the Receiver Operating Characteristic (ROC AUC) curve as well as the class-averaged balanced accuracy of the model predictions on the test dataset. All three classifiers were able to discriminate between the nuclei from different stages of cancer with similar efficiencies (Fig. S3a). Since LDA was faster, more interpretable and less subjective to hyper-parameter tuning, its discriminants were used for further exploratory analysis. The classification was stage specific (greater than 50%) for the majority of nuclei in the tissues (Figs. 3b, S3b) and most misclassifications occurred between related stages. For instance, nuclei from the normal and hyperplasia tissue have the highest class-wise accuracy and are rarely misclassified as one of the cancer tissues (Fig. S3b). These results indicate that the nuclear morphology and chromatin organization features can capture stage specific chromatin reorganization at the population level.

In order to understand how chromatin organization is changing during the progression of cancer, we obtained a pseudo-trajectory by building a diffusion map using the multivariate features of nuclei that were correctly predicted by the model (Fig. 3c)^29^. We identified three branches in this trajectory that were named Normal, DC (Ductal Carcinoma) and Invasive based on their constituents (Fig. S3c)^30^. Interestingly we find that the cells are ordered by increasing cancer stages. Nuclei from the normal tissue exclusively belonged to the Normal branch. In contrast, the nuclei from fibroadenoma and DCIS tissues constituted the DC branch. Most of the nuclei from ILC and IDC tissue belonged to the Invasive Branch (Fig. S3d). We also observed that there is a considerable overlap between the stages in the diffusion map, possibly reflecting the heterogeneity in the various clonal populations of cancer cells within a tumor. The appearance of the branch chiefly consisting of nuclei from intermediate stages, could be indicative of transient cancer state stages or the stromal cell recruitment and activation that occurs during DCIS.

The pseudo-trajectory indicates that there is a progressive reorganization of chromatin structures in the tissue population that occurs from normal to invasive cancer nuclei. Figure 3d summarizes how nuclear morphology and chromatin organization features change along the 3 branches of the trajectory. Chromatin condensation is high in the normal branch as indicated by high values of contrast and low values of homogeneity. Finally, we see nuclei with higher curvature values in invasive cancer tissues. Hence, there are specific changes in the nuclear shape and chromatin texture along each branch. We were particularly interested in the changes that occur along the normal to invasive path. This is explored in the next section.

### Single cell Mechano-Genomic Score that reflects breast cancer progression

To visualize nuclei in a TMA that are becoming tumorigenic, we next aimed to reduce the dimensionality of the chromatin feature space such that we obtain a score that is different for a nuclei from cancer and normal tissue. To that end, we trained a classifier (LDA) to distinguish nuclei obtained from normal breast and hyperplasia tissues (categorized as “normal”) from nuclei that were obtained from IDC and ILC tissues (categorized as "cancer"). This classifier has a classification accuracy of 89% on the test dataset (Fig. S4a). We next obtained the fraction of nuclei in each TMA correctly predicted as either normal or cancer. In normal tissues, over 90% of the nuclei were correctly predicted as normal. On the other hand, in cancer tissues the fraction of nuclei predicted as cancer was variable between TMAs but was always above 50% (Fig. S4b). This is expected as cancerous TMAs will contain a mixture of cancer and normal cells and the composition of these cells will be different for different tissues. Further, we also obtained the tissue level predictions based on the maximum number of predicted nuclear classes at the tissue level. This ensures that for a given TMA, if the majority of nuclei are predicted as “Normal”, the TMA will be classified as “Normal”. Remarkably all the tissues were correctly labelled in both the training and test datasets (Fig. S4a). This demonstrates that the cells in normal and invasive breast cancer tissues have different nuclear and chromatin organization features.

**Figure 4:**
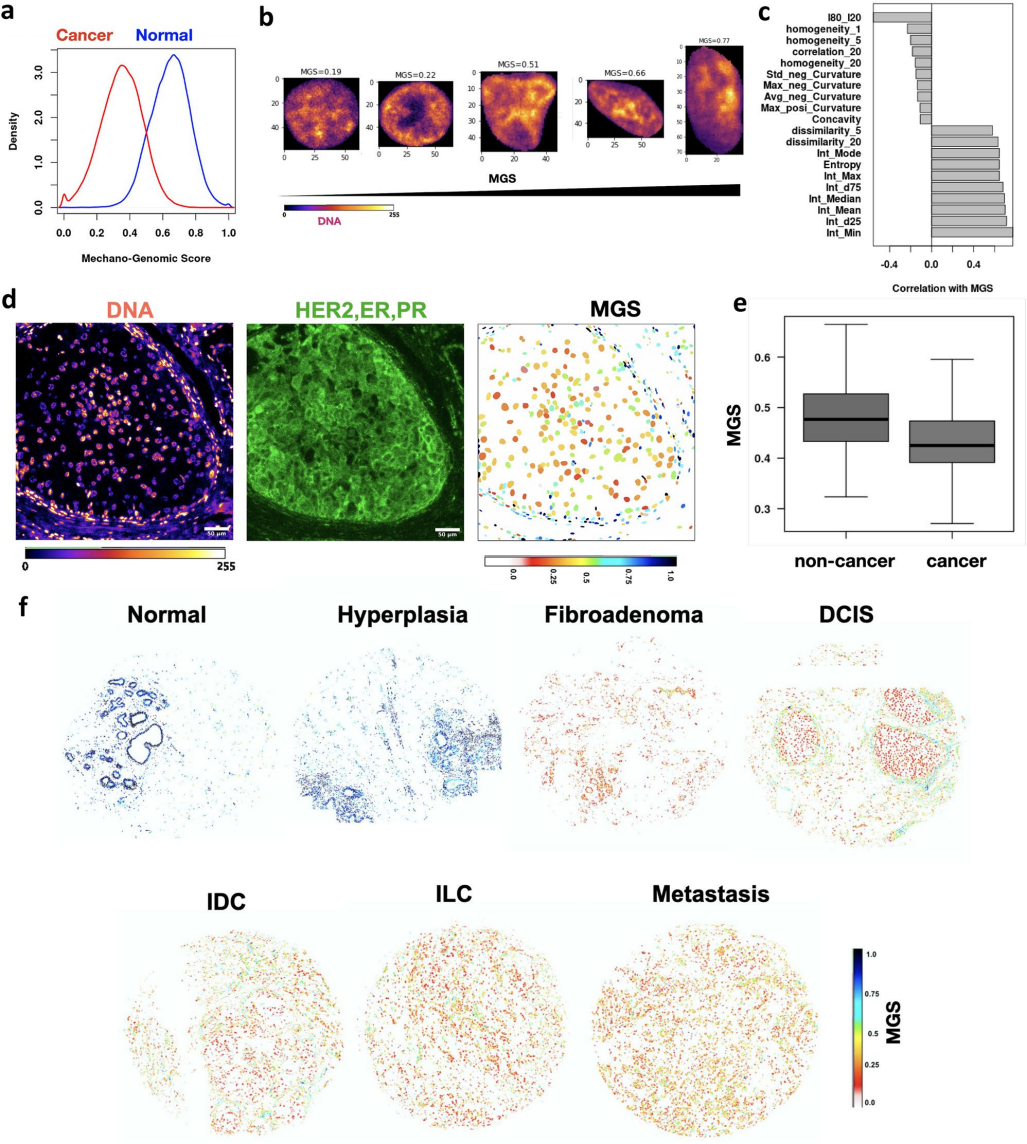
Single cell Mechano-Genomic Score that reflects breast cancer progression. (**a**) Probability density histograms of Mechano-Genomic Score (MGS) for nuclei from normal tissues (blue) and invasive cancer tissues (red) (Wilcoxon Rank Sum Test p-value < 0.001). (**b**) Representative images of nuclei stained for DNA (fire colors) with increasing MGS values (the top margin). (**c**) The top 10 features with the highest or lowest pearson correlation coefficients with MGS. Please note that the number following the features refers to the length scale of the GLCM texture features. (**d**) Micrograph of DCIS tissue sections stained for DNA and Biomarker (HER, ER and PR cocktail). Each nuclear boundary is colored based on the MGS of the corresponding nucleus. The color code is on the bottom right. (**e**) MGS of nuclei within and outside the cancer regions. Wilcox Test p-value < 0.0001 for E and F. n ~ 800. (**f**) Visualizing the MGS at single cell resolution for representative TMA of varying breast cancer stages. Each nucleus is colored based on its MGS. The color code is on the bottom right.

We next used the discriminant used by the classifier to define a Mechano-Genomic Score (MGS). This score for each nucleus would be a linear combination of multiple features that collectively describe its chromatin architecture. A high value of MGS corresponds to a healthy nucleus from normal breast tissue and lower values correspond to those from a cancerous tissue (Fig. 4a). Hence, MGS can be considered as a reflector of cell health. Consistently, the average MGS in a TMA was high in normal and hyperplasia tissues and low in other cancerous tissues (Fig. S4c). To understand the features that contribute to the MGS we obtained the Pearson correlation coefficients of all the features measured and the top interpretable features are summarised in Fig. 4c. Further, Fig. 4b. depicts cropped single nuclei arranged according to their MGS for visualizing these changes. These graphs illustrate that a healthy nucleus with high MGS has a smoother and more convex boundary (low Standard deviation of negative curvature and concavity), while the chromatin inside is denser (higher median intensity) and has rougher texture (higher dissimilarity and entropy and lower homogeneity). These are in line with a number of studies that have revealed that the organization of chromatin is significantly altered during tumor progression^25,27^.

In our TMA dataset, we only have the clinical annotation at the tissue level. To validate that a tumorigenic cell would have a lower MGS compared to a normal cell, ground truth data at the nuclear level is required. To that end, we selected three biomarkers routinely used by pathologists to characterise breast tumors: receptor tyrosine-protein kinase (HER2), estrogen receptors (ER) and progesterone receptors (PR)^31^. We selected two TMAs clinically annotated as DCIS that were outside the original dataset used in the previous results. This ensured that we would obtain a mixture of cancer and non-cancer cells in our images from a tissue our model has not seen before. We then performed immunofluorescence to label all three biomarkers along with the DNA (Fig. 4d) (see “Materials and methods”). From these images, we computed the MGS for each nucleus (Fig. 4d). We then established that the MGS was different between the nuclei within and outside cancer regions (Fig. 4e). Therefore, we conclude that the MGS can identify abnormal cells in the population.

The computation of MGS enabled us to visualize nuclei in a TMA that have abnormal chromatin organization. Figure 4f depicts this at the single cell level for whole tissue microarrays of varying stages of breast cancer. Normal tissues consist of nuclei with high levels of MGS which progressively decrease with breast cancer progression (Fig. S4c). Taken together, our results provide compelling evidence that supports the use of chromatin structure as a biomarker of tumorigenic states.

### Orientationally coupled regions are characteristic of early breast cancer states

In addition to the cancer cells, the tissue structure also undergoes changes during tumor progression. The cancer cells are known to display altered secretion of several biochemical factors such as TNF-α, TGF-β and interleukins^32,33^. These factors have been shown to alter the local microenvironment of the tissue leading to activation of stromal cells and increased matrix reorganization near the tumor which in turn aid in the progression of cancer. Therefore, we aimed to characterise the changes in tissue architecture during breast cancer progression using the nucleus as a probe. To this end, we focused on two key aspects: local crowding/cell density and long range tissue mechanics. Uninhibited proliferation of cancer cells as well as stromal recruitment by cancer cells are two factors that contribute to an increase in the density of cells in the local tumor microenvironment. Forces exerted by the expanding tumor mass on the stromal tissue and as well as increased extra-cellular matrix (ECM) deposition and aligned collagen fibers in the tumor stroma are some examples of the changes in the local tissue mechanics during tumor progression^1,2,34^.

To support our approach, we performed experiments to demonstrate the coupling between nuclear elongation and local ECM organization. We engineered a 3D in vitro tissue by embedding spheroids of Human Mammary Fibroblasts (HMF3A cell lines) in 3D collagen gels. After 24 h of culture, the fibroblasts extend and exert forces on the collagen matrix as seen by the local regions of ordered collagen around stretched fibroblasts with elongated nuclei (Fig. S5Ac). In order to relax the local tissue forces, we treated the gels with a specific protease that targets collagen: collagenase. Figure S5Aa shows time lapse images of the same field of view during mild collagenase treatment. As expected we saw that the fibroblasts become less extended and the nuclei are less elongated (lower Aspect Ratio) (Fig. S5Ab). To further check if these changes are occurring along collagen fibers, we fixed cells with and without mild collagenase treatment, stained for DNA, F-Actin and Collagen-I and imaged around regions of aligned collagen fibers, as shown in Figure S5Ac,d. Consistent with our live cell experiments, we find that cells and nuclei become less elongated after treatment Fig. S5Ae). In addition, we found that the nuclei become less elongated after the tissue tension was relaxed by local orthogonal sectioning of the collagen gel (Fig. S5Af,g). Further we co-stained collagen-1 and DNA in breast cancer TMAs. As seen in Fig. S5B, in regions where the collagen fibers are aligned, the elongated nuclei are also oriented along the same direction. In conclusion, our results show that nuclear elongation/Aspect Ratio can be used as a sensitive marker of local ECM organization.

**Figure 5:**
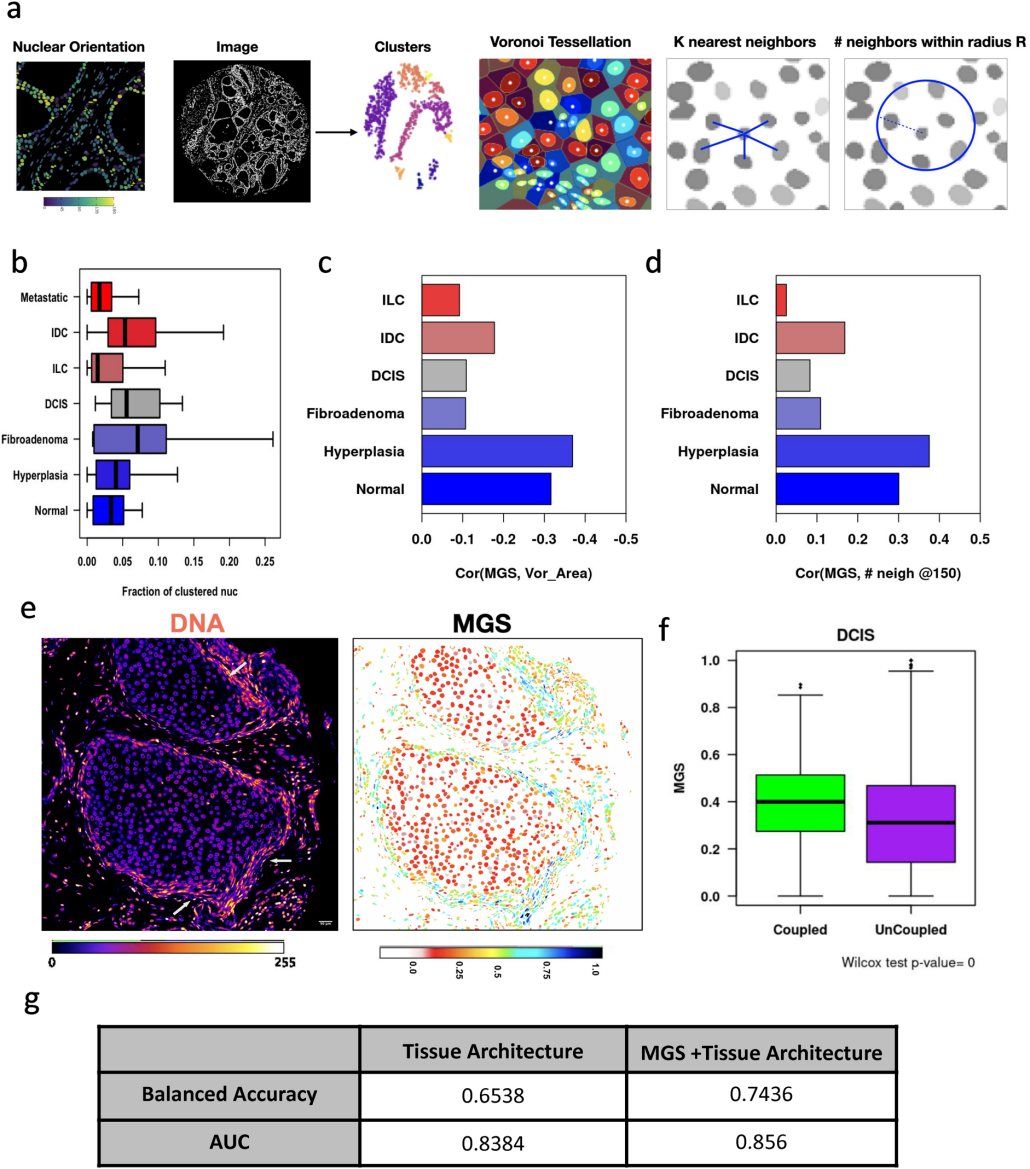
Orientationally coupled regions are characteristic of early breast cancer states. (**a**) Representation of the method used to identify orientationally coupled regions in the tissue. We first segment nuclei, obtain the angle that the major axis of the fitted ellipsoid makes with the *X* axis. We filter to obtain only elongated nuclei and then use their centroid and orientation as features for Density-based spatial clustering of applications with noise (DBSCAN). Each identified cluster is depicted in a different color. Local tissue density was calculated using multiple approaches namely, voronoi tessellation, distance to the kth nearest neighbour and number of neighbors in a given radius. (**b**) Fraction of clustered nuclei in the various stages of breast cancer. One way ANOVA indicated that there were significant changes in the means (p < 0.01). Correlation coefficient between MGS and Voronoi cell area (**c**) and number of neighbours in 40 µm radius (**d**). (**e**) Representative images showing the mechanically coupled regions (arrows) and the MGS of nuclei in Ductal Carcinoma in situ. (**f**) Mechano-Genomic Score (MGS) of clustered/coupled nuclei (green) and unclustered/uncoupled nuclei (purple) in Ductal Carcinoma In situ (DCIS) TMAs. (**g**) Tissue level predictions by Linear Discriminant Analysis using Tissue Architecture features with and without Mechano-Genomic Score.

Therefore, we measured features that characterise such long range forces in the TMAs using nuclei as a probe for tissue architecture. In Fig. 5a one can see regions in which all nuclei are pointing in the same direction indicating that these cells might be a part of the same local ECM structure. We identified these regions by performing density-based spatial clustering of applications with noise (DBSCAN) using the angles, and centroid coordinates of the elongated nuclei (see “Materials and methods”)^35^. We assume that the identified clusters represent spatial neighbourhoods where nuclei are orientationally coupled. We then calculated features that describe the clusters in tissues (Number of clusters, Fraction of nuclei clustered, area of clusters, relative distances between clusters etc.). In addition, we also measured tissue density via the following approaches: (1) the number of neighbors for each nucleus at radii ~ (5, 15, 25, 40 and 50) µm (2) distance between each nucleus and its kth nearest neighbor (k = 1, 3, 5, 10, 20) (3) Voronoi cell properties for each nucleus (area, perimeter and eccentricity). We find larger fractions of nuclei from Ductal Carcinoma In Situ, Fibroadenoma and Invasive Ductal Carcinoma are orientationally clustered (Fig. 5b). To further validate this, we have stained TMA of biopsies clinically annotated as normal, DCIS and invasive ductal carcinoma for collagen-1 and DNA and found that the intermediate DCIS stage has prominent collagen-1 enrichment (Fig. S5D). We further analysed the relationship as measured by the correlation coefficient between MGS with large scale architectural tissue features at the single nucleus level. In particular, we find that in normal tissues, nuclei with high MGS, have smaller voronoi areas and larger numbers of neighbours (Fig. 5c,d). Interestingly, in DCIS, the clustered/coupled nuclei have higher MGS indicating they are most likely normal cells (Fig. 5e,f). This suggests that it is the stromal cells that are undergoing the orientationally coupling in the early stages of cancer as suggested by many studies^17,22^. These results further highlight a relationship between the single cell MGS and tissue architectural features.

Further, in order to compare the discriminatory potential of these tissue architecture features and the mechano-genomic score, we obtained distribution characteristics of these features for each TMA (mean, dispersion, skewness, kurtosis, etc..) and set up a classification task. We used 80% of the TMAs for training and kept 20% for validation. The results for the test dataset are tabulated in Fig. 5g. The balanced accuracy for tissue classification is 65.4% when using only tissue architecture features but increases to 74.4% when MGS are included. Hence, while the tissue architecture features can discriminate between breast cancer stages, having the mechanogenomic score increases the efficiency significantly. Collectively our results highlight that image-based nuclear features can be used as a probe to capture the local reorganization of the tumor niche at the cellular scale as well as the tissue scale during the progression of cancer.

## Discussion

Tumor initiation and progression is driven by both intracellular changes within the tumor cells as well as the biophysical properties of the tumor microenvironment. The dynamic interplay of the mechano-chemical signals between the tumor and the stromal cells shape the local tumor microenvironment that is critical to tumorigenesis, including initiation of metastasis. Recently, there have been multiple studies that have revealed how the mechanical microenvironment regulates metastasis^36^. For most breast cancer types, stiffness of the tumor is higher than neighboring normal tissue and is highly correlated with cancer progression and metastasis^37,38^. The stiffening of tumor stroma can primarily be attributed to deposition and remodeling of ECM by the activated fibroblasts in the tumor stroma^36,39^. These activated fibroblasts exert long-range contractile forces that reshape the tissue architecture to promote metastasis^11,40,41^. The recent attempts to characterise the mechanics of tumor progression including imaging methods have revealed the alterations to the local-mechanical signatures of tissues during tumour progression^42^. In addition, several engineered 3D tissue models have demonstrated that the tumour microenvironment is held under mechanical tension particularly at the site of invasion^34,43,44^. Since recent studies have shown that the cell membrane is intimately linked with the nucleus, we suggest that alterations in the long range mechanical environment should reflect at the single cell chromatin structural changes. However, current tumor diagnostic methods using imaging, sequencing, and machine learning have focused on robustly classifying tumor stages using the biochemical signatures of the tumor microenvironment and have largely missed the mechanical coupling within the tumor microenvironment as the cancer progresses. Towards this end, developing a high resolution imaging based method to detect features that reflect nuclear morphometric and chromatin condensation patterns could provide robust characterization of cell state changes that occur during tumor progression. Further, since nuclear mechanics is tightly regulated by cellular tension^45^, we can use the nucleus as a probe to study the long range mechanical coupling within tissues.

In this paper we used patient derived tissue biopsies of various stages of breast cancer to decipher the cross-talk between tumor cells and stromal cells by building single cell chromatin maps. While H&E stained tissues combined with immunofluorescence have been the gold standard for histopathology, the contrast and information of genomic packing is superior when using DNA binding dyes such as Hoescht or DAPI. Since these dyes directly intercalate with the DNA structure, the resulting fluorescent image is a direct reflection of chromatin compaction. As a result, we show that staining the DNA with Hoescht significantly improves the accuracy of classifying cell states using our integrated image based machine learning pipeline. One drawback of using H&E stained tissues is the variability in the staining and this can be mitigated by using Hoechst. We were also able to use a linear classifier to discriminate between different stages of cancer. In order to map the structural changes in the features used by our classifiers during cancer progression, we built an unsupervised diffusion model of the correctly predicted nuclei. Such approaches have been widely used in single-cell sequencing studies^46,47^, and here we apply a variant of that for chromatin images of tissue biopsies at different tumor stages. Our trajectory had three branches, namely the normal branch which consisted of nuclei from normal and Fibroadenoma tissues, the DC branch which consists of the nuclei from Ductal Carcinoma In Situ and the metastatic branch with consists of nuclei from Invasive Lobular Carcinoma, Invasive Ductal Carcinoma and Metastatic tissues. We found that there is a progressive loss in the contrast of the chromatin organization and more rounder curvatures in nuclei from normal to invasive cancers. These are in line with a number of studies that have revealed that the spatiotemporal organization of chromatin is significantly altered during tumor progression.

Using the imaging based features that are important for discriminating the normal and invasive stages of cancer we provide a mechano-genomic score for single cells. Such a score constructed by dimension reduction provides a fingerprint of the cellular state of every nuclei. We further validated our score by demonstrating that it could distinguish cells expressing standard biomarkers such as HER2, ER and PR^31^. Since the ECM organization at the tissue scale reflects at the individual nuclei and chromatin state, the MGS combined with nuclear orientations directly provide spatio-temporal neighbourhoods of the orientationally coupled cells. Notably, in Ductal Carcinoma In Situ, we see low MGS in the ducts and the surrounding stromal cells have high MGS and the stromal cells are orientationally coupled around the ductal regions. This is indicative that these clusters could be composed of highly contractile stromal cells and as a result of local tissue tension and higher matrix alignment as posited by multiple studies^36,40^. Such analysis provides direct snapshots of spatial interactions of functional hubs around important tissue landmarks.

Collectively, our imaging based mechano-genomic scoring of cells not only enables the classification of tumor stages, but since the features are interpretable, it also allows us to gain mechanistic insights on the alterations to the mechanical environment of tissues during tumor progression. MGS is highly sensitive and can detect small subsets of abnormal cells in a heterogenous microenvironment. While, in this study we have used TMAs, using whole slide images of tissues will provide more information on the heterogeneity of the tumor and the stromal regions. The advantage of using chromatin patterns as a functional biomarker of a cell state provides unique possibilities without the need to perform single cell sequencing or proteomics of complex heterogeneous tumor microenvironments for disease diagnostics.

## Materials and methods

### Staining of tissue microarray

Source: All experiments were performed in accordance with relevant guidelines and regulations at National University of Singapore (NUS). Hematoxylin and Eosin (H&E) stained and unstained tissue microarray (TMA) slides (BR1003A, BR301A and BR2082B) were procured from US Biomax (US Biomax, Inc., Derwood, USA). These contain breast biopsy samples from healthy as well as cancer patients of varying stages. While images of the H&E-stained samples were provided by US Biomax, the unstained slides were stained with Hoechst and imaged in-house. For more information please visit (https://www.biomax.us/).

The slides were immersed in Xylene twice (5 min each) to completely remove the paraffin embedding. Subsequently, successive rehydration steps were performed with decreasing concentrations of Ethanol (twice in 100% ethanol for 3 min each, twice in 95% ethanol for 3 min each, once in 80% ethanol for 3 min, once in 70% ethanol for 3 min and once in 50% ethanol for 3 min). The slides were then washed in PBS twice for 3 min each and incubated with Hoechst solution for 10 min. Finally, the slides were washed with PBS, embedded in mounting media and sealed with a coverslip.

To perform immunofluorescence, TMAs were incubated with antigen retrieval solution according to the manufacturer's instructions at 98 °C for 30 min. Following this, the tissues were incubated in a blocking solution (10% goat serum in PBS) for 20 min. A mixture of cancer markers was obtained by simultaneously incubating the tissue with three antibodies (HER2 ab237715, ER ab32063 1:200, PR ab32085 1:200). Collagen-1 was stained using the primary antibody ab6308 (1:200). After this, the tissues were incubated in the primary antibody solution overnight at 4 °C. The slides were then washed three times with 1xPBS and the incubated with the secondary antibody solution (Alexa Fluor Goat anti-Rabbit 647) and DAPI for 1 h. Finally, the slides were washed with PBS, embedded in mounting media and sealed with a coverslip.

### Imaging of tissue microarray slides

Images of Hoechst-stained tissues were acquired on the TissueFAXS Slide Scanner by TissueGnostics. Stage calibration was performed before each imaging session and the autofocus function was used for maintaining focus throughout the session. A 10X air objective was used for performing the initial fast scan in order to delineate the biopsy regions on the slides. After manual verification and adjustments, the final acquisition was performed using a 40X air objective.

### Segmentation of individual nuclei from large images

Our goal here was to automatically segment well-imaged nuclei from images. The raw images are normalized and converted to grayscale ones with the brightest pixel being assigned a value of 1 and the dimmest pixel being assigned a value of 0. The image is then blurred using a Gaussian filter and thresholded according to Otsu’s method. The remaining noise is removed from the binary image using a median filter. Subsequently the distance transform of the image is calculated, and watershed is used to demarcate individual nuclei on the mask. Finally, the mask is used to segment and crop out every nucleus from the grayscale image and each is analysed as described below. This was performed in MATLAB and QuPath^48^. For dense tissue images, a StarDist model was used. Briefly, it uses a convolutional neural network to predict nuclear instances as star-convex polygons^19^. To train and test the network, we used images from the 2018 DSB nuclear segmentation challenge, in addition to 1000 nuclei from 10 different TMAs for which the ground truth labels were manually generated in house. Our model has a segmentation sensitivity of 82% on the validation dataset which is inline with the other top performing models^20^.

### Single-nuclear feature extraction

A custom written program was used to first normalize the intensity and then extract a large number of morphological and textural features from each segmented nucleus. These features belonged to four broad groups, namely, global morphological features, nuclear boundary features, global intensity features and intensity distribution features (Fig. 1). The list of primary features include area, perimeter, aspect ratio, peripheral distribution index, separation between the centre of mass and the centroid, heterochromatin to euchromatin ratio, the mean, median, standard deviation and mode of the normalized intensity, relative concavity, caliper diameters, entropy of intensity distribution, boundary curvatures and moment invariants (Supplementary Table S1). In addition, compound features such as the fractions of the perimeter which are positively and negatively curved were also calculated.

### Supervised classification

All classification tasks were carried out in R^49^. The dataset was filtered to obtain nuclei whose area is between 2 and 300 squared microns with a normalized median intensity of 10 and a standard deviation of intensity of at least 2. This filtration step ensured that only interphase nuclei that were in focus were used for further analysis. This dataset was randomly sampled to generate training and test datasets. The training dataset consisted of around 7200 nuclei sampled equally from 80% of the TMAs for each cancer stage. The rest were assigned to the test dataset. Since we did not have ground-truth labels at the single cell level, cells were assigned the clinical annotation of their tissue. Principal component analysis (PCA) was then performed on the scaled data to generate a set of orthogonal features, of which 50 were used for classification by a linear discriminant classifier (explained 90% of the variance). An artificial neural network was designed with two hidden layers and trained using the first 50 principal components of the training dataset. The random forest classifier was trained using 2000 estimators with a maximum depth of 7. We used a fivefold cross-validation to determine model generalizability. Performance of the models was determined based on the area under the receiving operating characteristic curve and class-averaged balanced accuracy on the test dataset.

### Diffusion map

We built a diffusion map to construct the pseudo-trajectory and used a k-nearest neighbors graph (k-NN graph) for visualization to detect branches. We used the Linear discriminants of 200 cells per cancer stage that were randomly sampled from nuclei that were correctly predicted by LDA. The width of the gaussian kernel used was 0.8 and was obtained using heuristic estimation and euclidean distance was used as a metric. This was carried out in R using the destiny and complexheatmap packages.

### Identifying orientationally coupled regions

Orientationally coupled regions are assumed to be regions in which elongated nuclei are pointing in the same direction. This indicates that these nuclei are all experiencing the same tension and thereby are coupled. We performed Density-based spatial clustering of applications with noise (DBSCAN)^50^ by using the angles, and centroid coordinates of the elongated nuclei. We found that the median distance to the and the median difference in the orientation angles with the kth nearest neighbour stopped increasing after 15 neighbours (Fig. S5Ca,b). Further, the number of clusters found was maximum for a neighbourhood radius of 400 (Fig. S5Cc). Therefore, we used a neighborhood radius ε of 400 pixels and minimum size of a cluster to be 15 nuclei. This was performed using sk-learn’s DBSCAN.

### Identifying cancer regions using biomarker expression

We identified cancerous regions in tissues by performing intensity based thresholding of the biomarker expression. Cells within such cancerous regions were identified to be cancer cells. To obtain the cellular levels of these proteins, we expanded the nuclear boundary by 5 μm or until it touched another nuclear boundary, and then measured the protein levels in this expanded 'cell' region.

## Data availability

The code used is available at https://github.com/GVS-Lab/genomic-scoring-breast-cancer-progression.git

## Supplementary Information

Supplementary Information.
