# Supplementary material for: Single cell imaging-based chromatin biomarkers for tumor progression
Source: Sci Rep. 2021 Nov 29;11:23041. doi: 10.1038/s41598-021-02441-6 (PMC8630115; doi:10.1038/s41598-021-02441-6)
Supplement: Supplementary file 1 — Supplementary Information. [file 41598_2021_2441_MOESM1_ESM.pdf]

Supplementary Information

Supplementary Materials and Methods

Cell culture

Unless otherwise mentioned, all the cell lines were cultured in high-glucose Dulbecco's Modified Eagle Medium (DMEM) (Gibco; Life Technologies) supplemented with 10% (vol/vol) Fetal Bovine Serum (FBS) (Gibco; Life Technologies) and 1% penicillin-streptomycin (Penstrep)(Gibco; Life Technologies) at 37 °C and 5% CO2 in humid conditions. The cells were rinsed with 1% Phosphate-buffered saline (PBS), fixed with 4% Paraformaldehyde (PFA) for 10 minutes, washed with PBS and incubated with 1 μg/ml Hoechst for 20 mins before imaging.

CellTracker Green (ThermoFisher Scientific) was used for labelling the cytoplasm of HMF, HME and BJ cells (pseudocolours were applied to make them red, green and blue respectively). Labelling was conducted by incubating the live cells with 3 μM in serum free media for 30 mins as per the manufacturer's protocol. Thereafter, the cells were rinsed twice with PBS and cultured in complete culture media as described above.

Conditioned media

Conditioned media was prepared using actively proliferating HME, MCF10A and MDAMB231 cells. Fresh media (high-glucose DMEM with 10% FBS and 1% Penstrep) was supplied when the cultures reached around 70% confluency and was collected back after 24 hours. The collected media was syringe-filtered through 0.45μm pores to remove cell debris.
The MCF10A and MDAMB231 conditioned media were diluted to 20% and 60% in HME conditioned media and these were subsequently used to incubate HMF cells for 3 hours and 3 days. 100% HME conditioned media was used as control. The cells were fixed and stained as described above.

Imaging of cell cultures

A 40X water objective with numerical aperture of 1.25 was used on a NikonA1Rsi confocal microscope to image the cell cultures. This configuration was chosen in order to obtain the optimum resolution while allowing for large-field imaging. The pin-hole was kept open (3 airy unit) and single plane imaging was carried out. The laser power was adjusted such that the whole range of the detector was used without any saturation. The pixel size was set to 0.12μm and the automatic stitch function was used to image areas of 2mm by 2mm.

In vitro 3D tissue culture

HMF3A fibroblast cells were trypsinized from a 70% confluent culture and 50,000 cells were added to a 1 mg/ml rat tail collagen I (ThermoFisher Scientific) solution which was neutralized with 0.1 N NaOH. This solution was allowed to gel at 37°C for 3 hours, after which 0.5 ml of culture media was added and the samples were kept for 24 hours.Nuclei were stained using Hoechst and imaged.

Supplementary Tables

Supplementary Table 1: Feature description

Feature	Description	Feature category	Feature type
Projected Area	Projected area of the nucleus	Global morphology	Morphology
Aspect Ratio	$\frac{Major\ axis}{Minor\ axis}$	Global morphology	Morphology
Perimeter	Perimeter of the nucleus	Boundary	Morphology
Shape Factor	$\frac{Perimeter^2}{4\pi\ x\ Area}$	Global morphology	Morphology
Relative Concavity	$\frac{Convex\ area - area}{Convex\ area}$	Global morphology	Morphology
Centre-Centroid Mismatch	Distance separating the centroid of the nucleus and the centre of mass of the chromatin intensities	Intensity distribution	Mixed
Entropy	Statistical measure of randomness in the distribution of intensities given by -sum(p.*log2(p)), where p contains the normalized histogram counts.	Intensity distribution	Intensity
Mean, median, SD and mode of Normalized Intensities	Frequency analysis of the normalized pixel intensities	Global intensity	Intensity
Peripheral distribution index	$PDI = \frac{M\_actual}{M\_ref} = \frac{\Sigma(r_{(x,y)}^2 * I_{(x,y)})}{\Sigma(r_{(x,y)}^2 * <I>)}$<br>$r_{(x,y)}$: Distance of pixel (x,y) from the centroid of the nucleus<br>$I_{(x,y)}$: Intensity of pixel (x,y)<br>$< I >$: Average intensity of the nucleus	Intensity distribution	Mixed
Heterochromatin euchromatin ratios	Heterochromatin pixels are defined as pixels having intensities greater than the 80th percentile of all the pixels of that nucleus. Euchormatin pixels are defined as those with intensities below the 20th percentile of the nucleus.	Global intensity	Intensity

Caliper diameters	The largest and smallest separation between two parallel plates which can fit the nucleus without losing contact.	Boundary	Morphology
Local curvatures	The nuclear boundary was smoothened using a moving average function with a span of 2 µm. The radius of curvature was then calculated for each pixel using stretches of 1 µm on each side. The radius was taken as negative if the centre of the circle was in the direction opposite to the centre of the nucleus.	Boundar y	Morpholo gy
Boundary distances	The distance of each point on the boundary from the nuclear centroid.	Boundar y	Morpholo gy
Moments	Hu moment invariants	Intensity distributi on	Mixed

a.

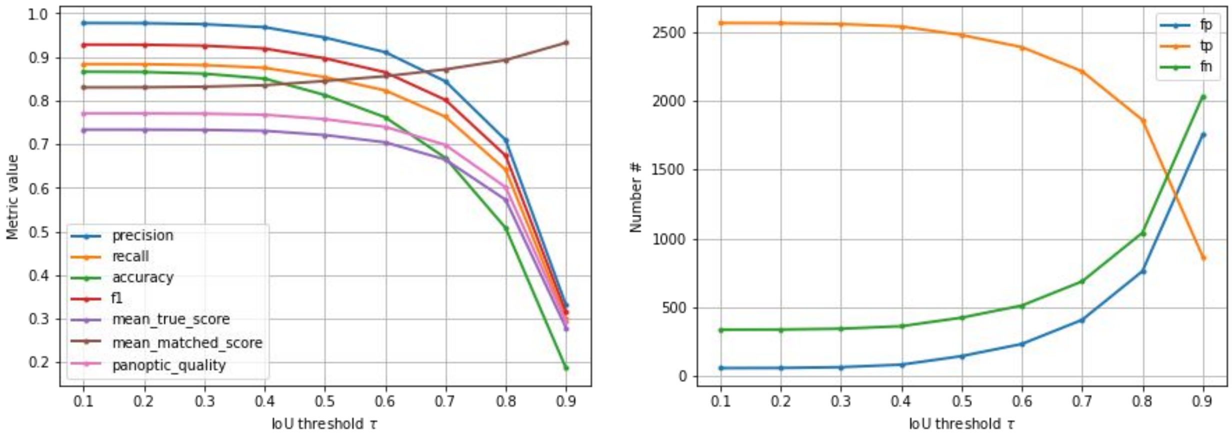

b.

Model	Recall at 0.7 IoU	Average F1	Accuracy
Ours	0.8233	0.8459	0.7583
StarDist pretrained	0.6916	0.7269	0.5249
2018 DSB Winner*	0.7762	0.7120	NA

Figure S1A: Nuclear Segmentation

a. Accuracy curves depicting the model performance b. Table summarizing the F1, Recall and Accuracy of of various models.

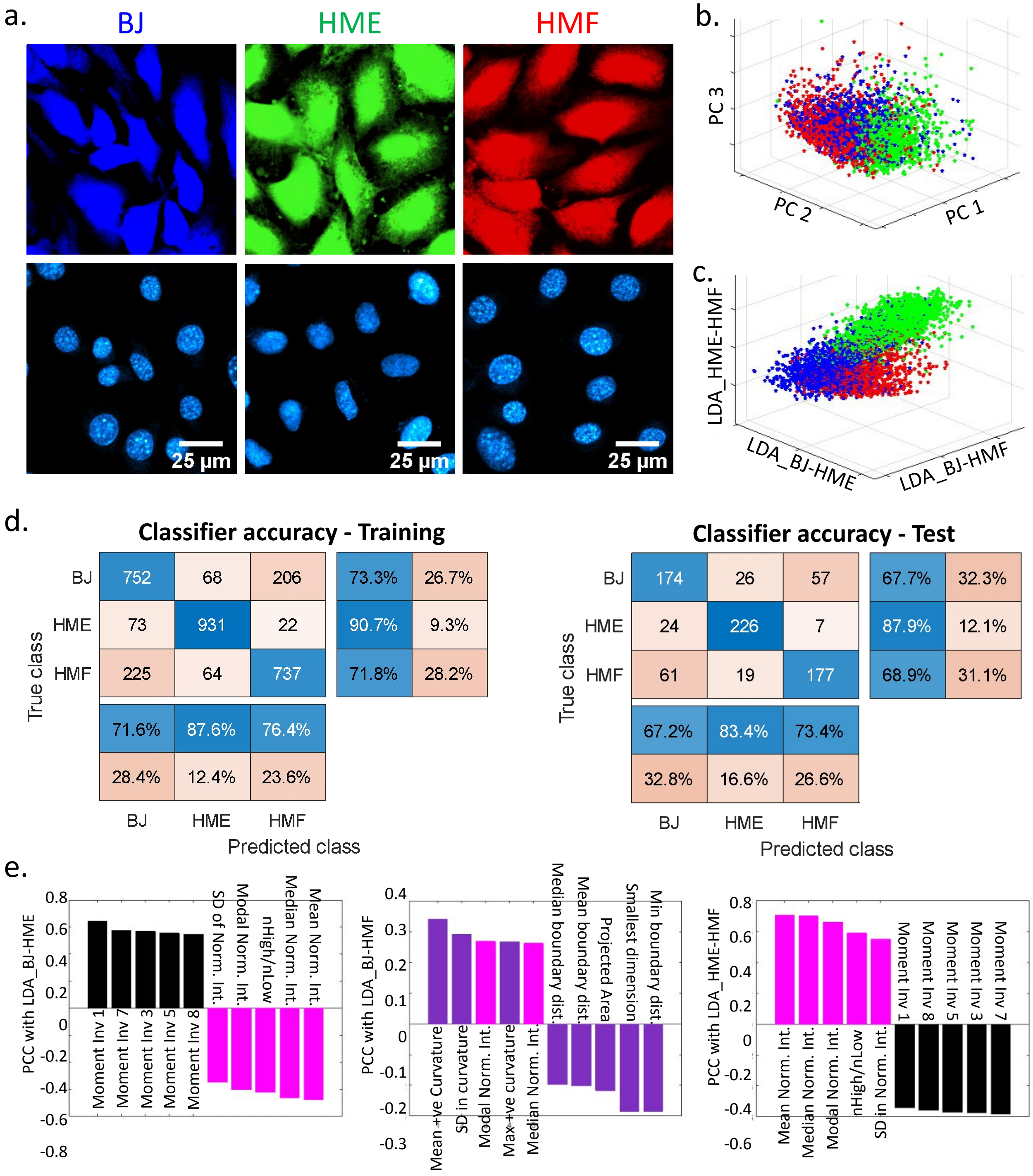

Figure S1B. Classification of three non-cancerous human cell lines based on single cell chromatin organization features

a) Representative images of BJ, HME and HMF cells stained with CellTracker Green (top row) and DAPI (bottom row). Different pseudo-colours have been applied on the CellTracker Green channel of the three cell lines. b) The first three principal components of the single cell chromatin organization data. Each dot represents one nucleus. Blue: BJ nuclei, Green: HME nuclei and Red: HMF nuclei. d) Confusion matrices depicting the training (left) and test (right) performances of the linear discriminant classifier in classifying nuclei from the different cell lines according to their chromatin organization features. e) Top ten interpretable features most correlated with the linear discriminant axes (left: BJ-HME axis, middle: BJ-HMF axis and right: HME-HMF axis).

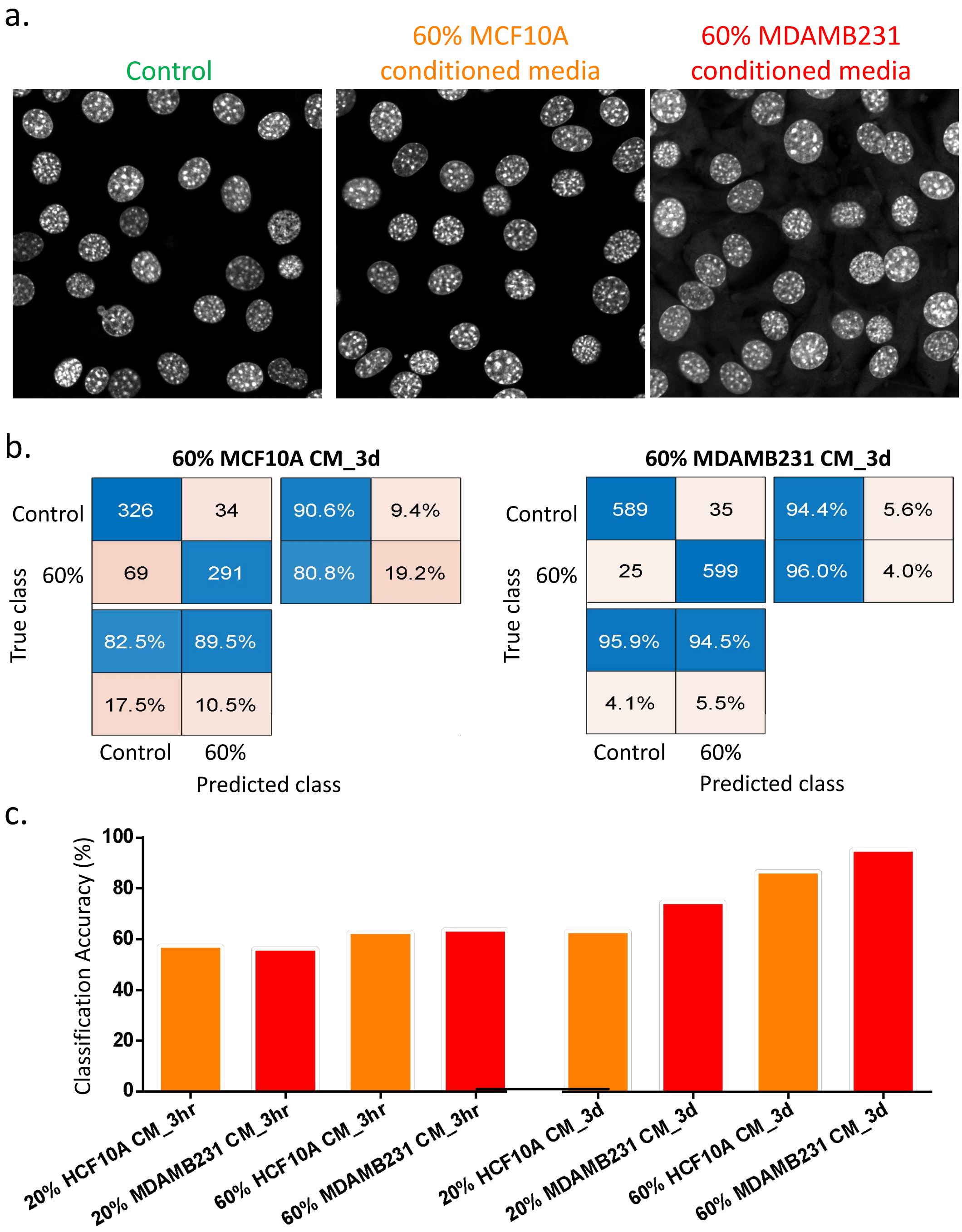

Figure S1C. Sensitivity of chromatin organization features of normal cells to exposure to the secretome of cancerous cells

a) Representative nuclei (DAPI) images of HMF cells incubated in control conditioned media (left), 60% MCF10A conditioned media (middle) and 60% MDAMB231 conditioned media (right). b) Classification accuracy of linear discriminant classifiers in discriminating between nuclei of HMF cells treated with control conditioned media and those treated with either 60% MCF10A conditioned media (left) or 60% MDAMB231 conditioned media (right). c) Accuracies of linear discriminant classifiers in discriminating between nuclei of HMF cells treated with control conditioned media and those treated with MCF10A and MDAMB231 conditioned media of different concentrations and for different durations.

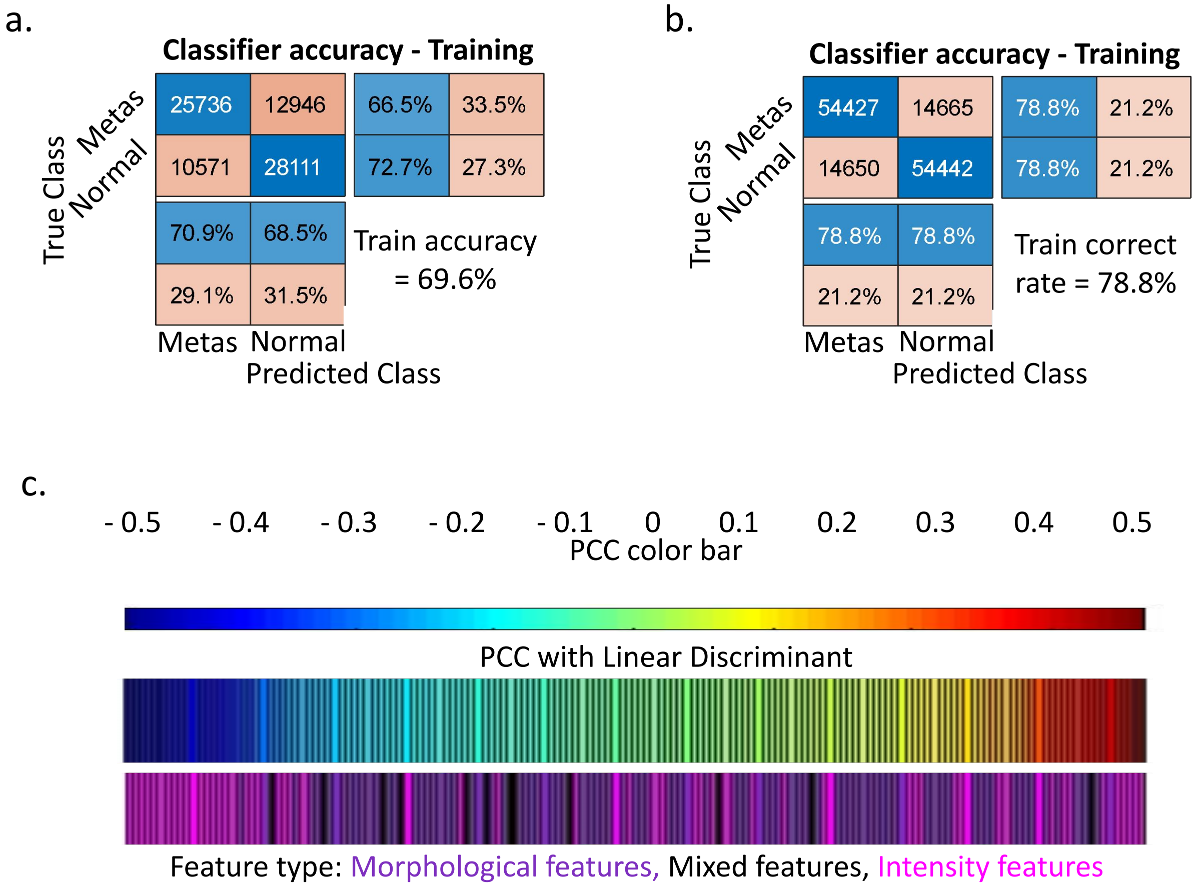

Figure S2. Hoescht staining improves the sensitivity to discriminate tumor progression

Confusion matrices depicting the performances of the linear discriminant classifier in classifying nuclei from normal and metastatic tissues according to their nuclear features in H&E-stained tissues(a) and Hoescht stained tissues (b). (c) Correlation between nuclear features and the linear discriminant axis for the H&E-stained nuclei. The features have been colour-coded and grouped into morphological features, mixed features and intensity features.

a.

Metric	LDA	RF	ANN
ROC AUC	0.8134	0.7933	0.7112
Balanced Accuracy	0.6680	0.6517	0.6241

b.

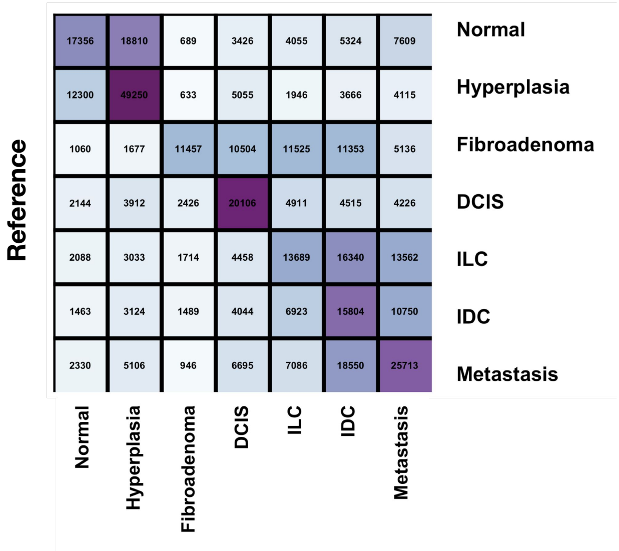

c.

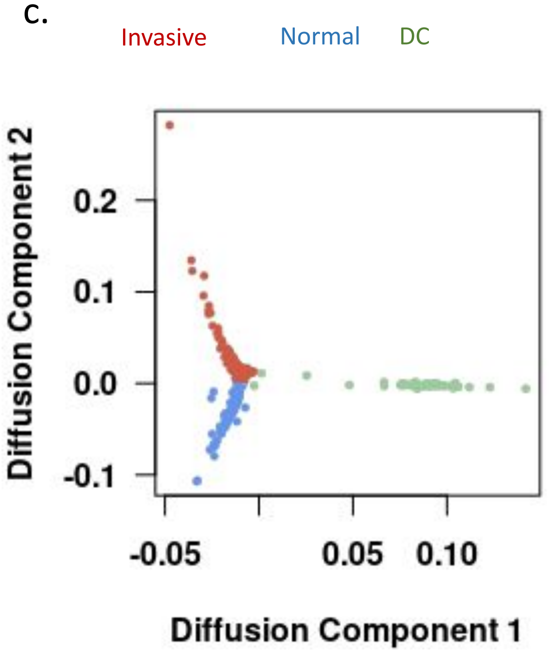

d.

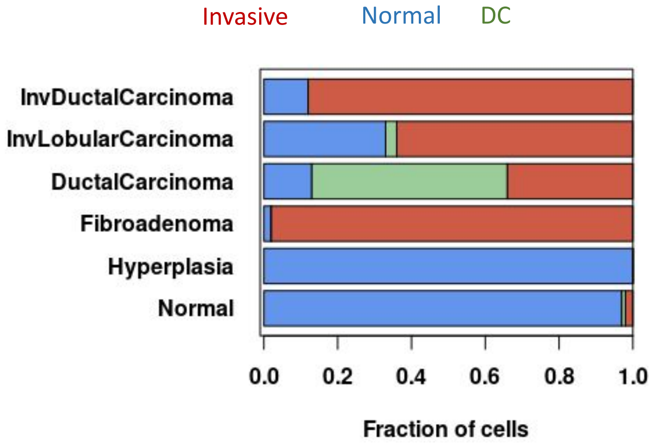

Figure S3. Building a trajectory of chromatin reorganization associated with Breast cancer progression

a) Discriminatory potential of the various models to distinguish nuclei from different stages of cancer in the test dataset. b) Confusion matrix depicting the classification results on the test dataset. c) Identifying branches on the Diffusion Map of nuclei from various stages of cancer. Each dot represents one cell. d) The branch membership fraction of the cells from the various cancer stages. The color code is displayed along the top margin for (c) and (d)

a.

	Nuclear level		Tissue level	
Metric	Training	Test	Training	Test
Accuracy	0.8727 +/- 0.0085	0.8919	1	1
ROC(AUC)	0.9478 +/- 0.005	0.9603	1	1

b.

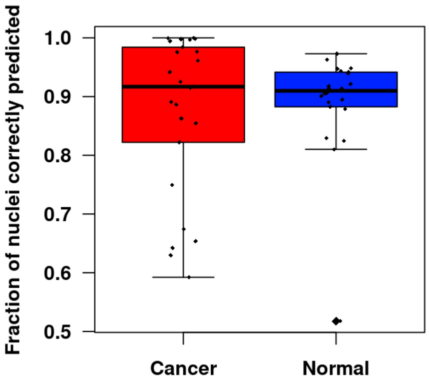

c.

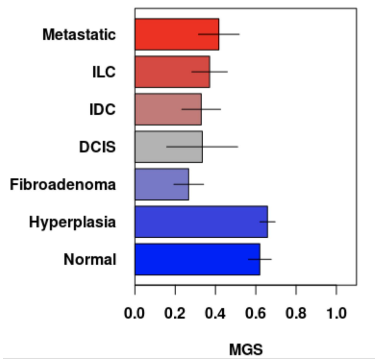

Figure S4. Single cell Mechano-Genomic Score that reflects breast cancer progression

a) Confusion matrices of the LDA discriminating between nuclei from normal and invasive cancerous tissue on the test dataset. b) Fraction of nuclei that are correctly predicted in the normal (blue) and cancer (red) tissues by the classifier. c) The mean MGS of TMAs across breast cancer stages. The error bar represents the standard deviation across TMAs. One way ANOVA indicated that there were significant changes in the means (p<0.01).

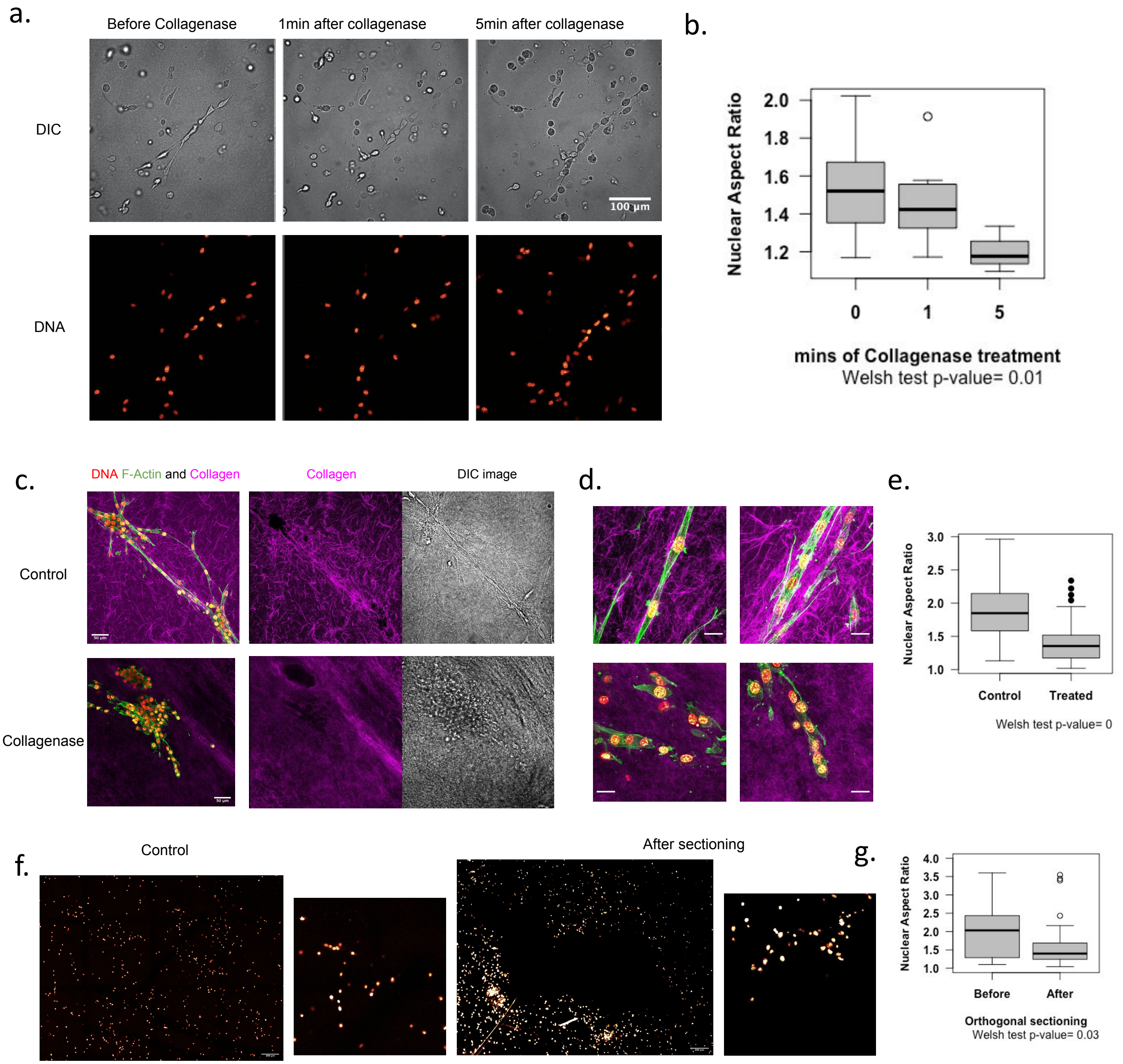

Figure S5A: Orientationally coupled regions are characteristic of early breast cancer states

a) HMF3A fibroblasts in 3D collagen gel imaged for DNA (red) and Differential Interference Contrast (DIC) image. Upper panel contains a representative DIC images of cell shape changes and the bottom panel contains Hoescht stained nuclei of the corresponding cells during mild collagenase treatment. Scale bar is 100 microns. b) Boxplot depicting the changes to the nuclear Aspect Ratio during treatment. The comparison is done between 0 and 5 mins. c) HMF3A fibroblasts in 3D collagen gel stained with DNA (red), F-Actin (green) and Collagen-I (Magenta) and Differential Interference Contrast (DIC) image. Upper panel contains a representative image under control conditions and the Bottom panel contains a representative image after mild collagenase treatment. Scale bar is 50 microns. d) Zoomed in regions of local tension as identified by the oriented collagen fibers as well as cells under control conditions(upper panel) and after mild collagenase treatment (bottom panel).Scale bar is 20 microns. e) Aspect ratio of nuclear with and without collagenase treatment. f) Large field view of nuclei before and after sectioning. Scale bar is 200 microns. Zoomed in regions are on the right. The void in the middle denotes the region of the section. g) Boxplot depicting the changes to the nuclear Aspect Ratio after sectioning.

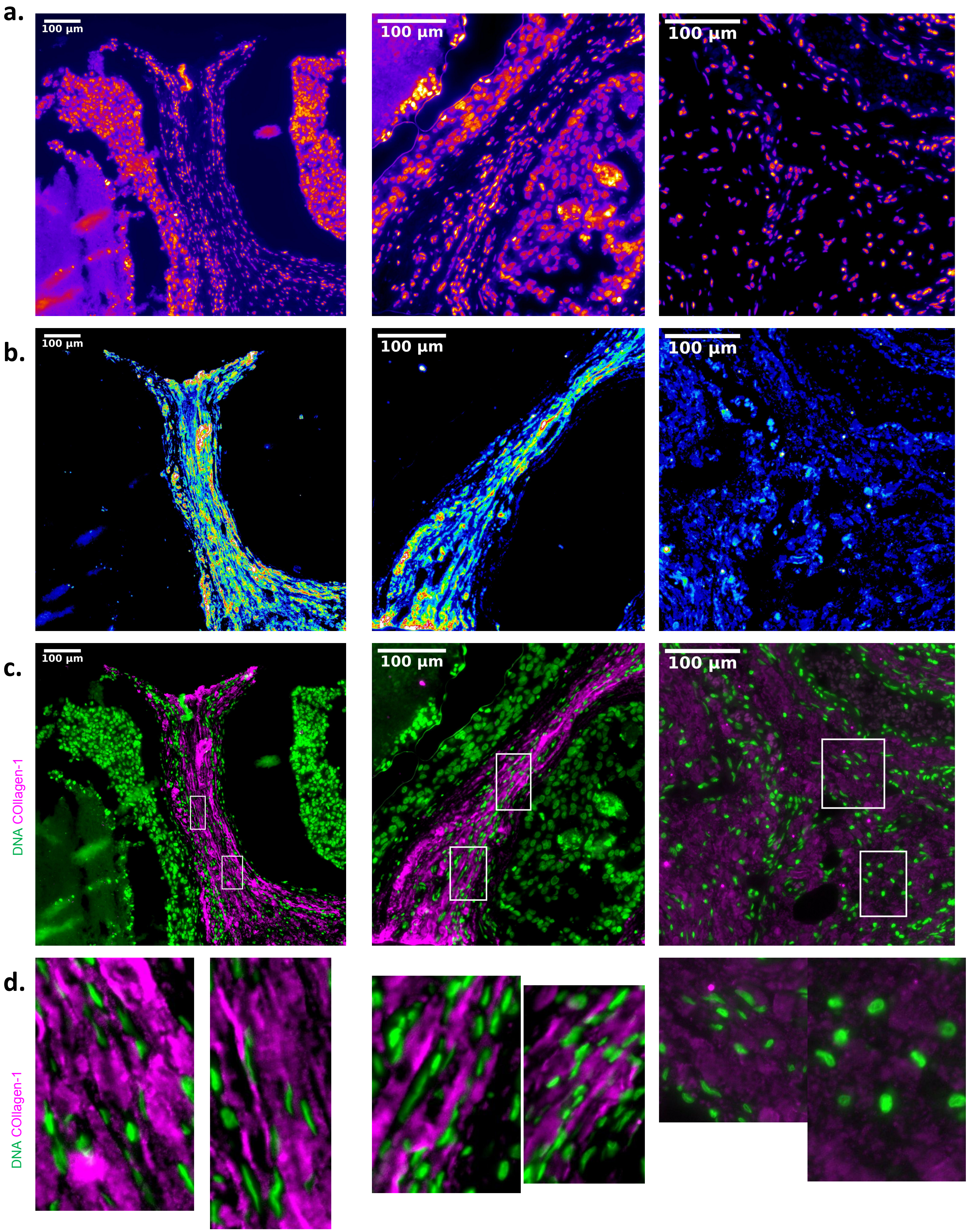

Figure S5B: Orientationally coupled regions are characteristic of early breast cancer states

Different regions from a DCIS TMA stained for DNA (a) and Collagen-1 (b). (c) Merged image of DNA (green) and Collagen-1 (Magenta). Scale bar is 100 microns. White boxes represent regions that are zoomed in (d)

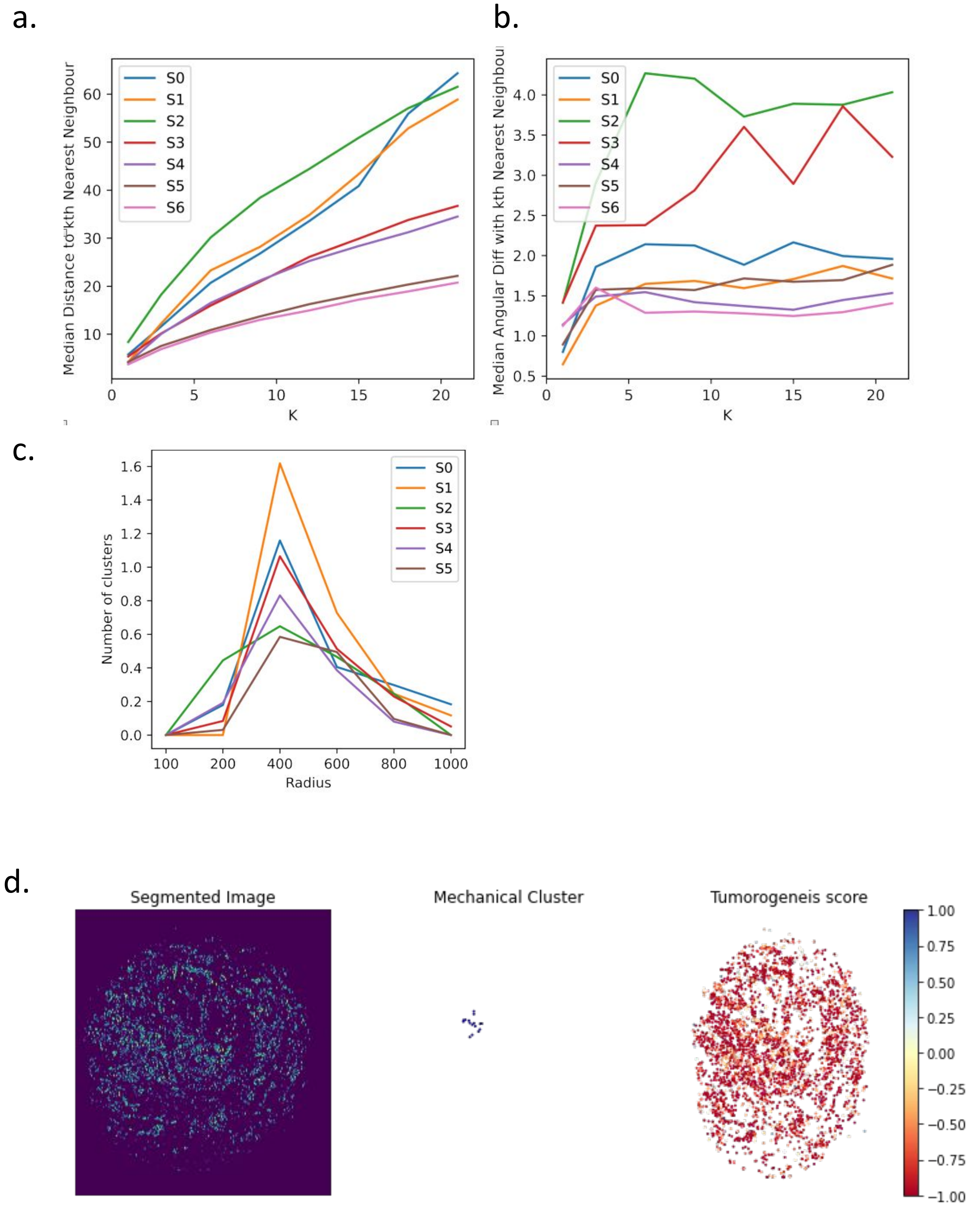

Figure S5C. Orientationally coupled regions are characteristic of early breast cancer states

a) Median distance from the Kth nearest neighbour (pixels) b) The median angular difference between a nuclei and its kth nearest neighbour. c) Number of clusters found as a function of increasing radius using DBSCAN d) Representative image of a metastatic TMA showing the image, segmented nuclei , Mechanical clusters found and the MGS.

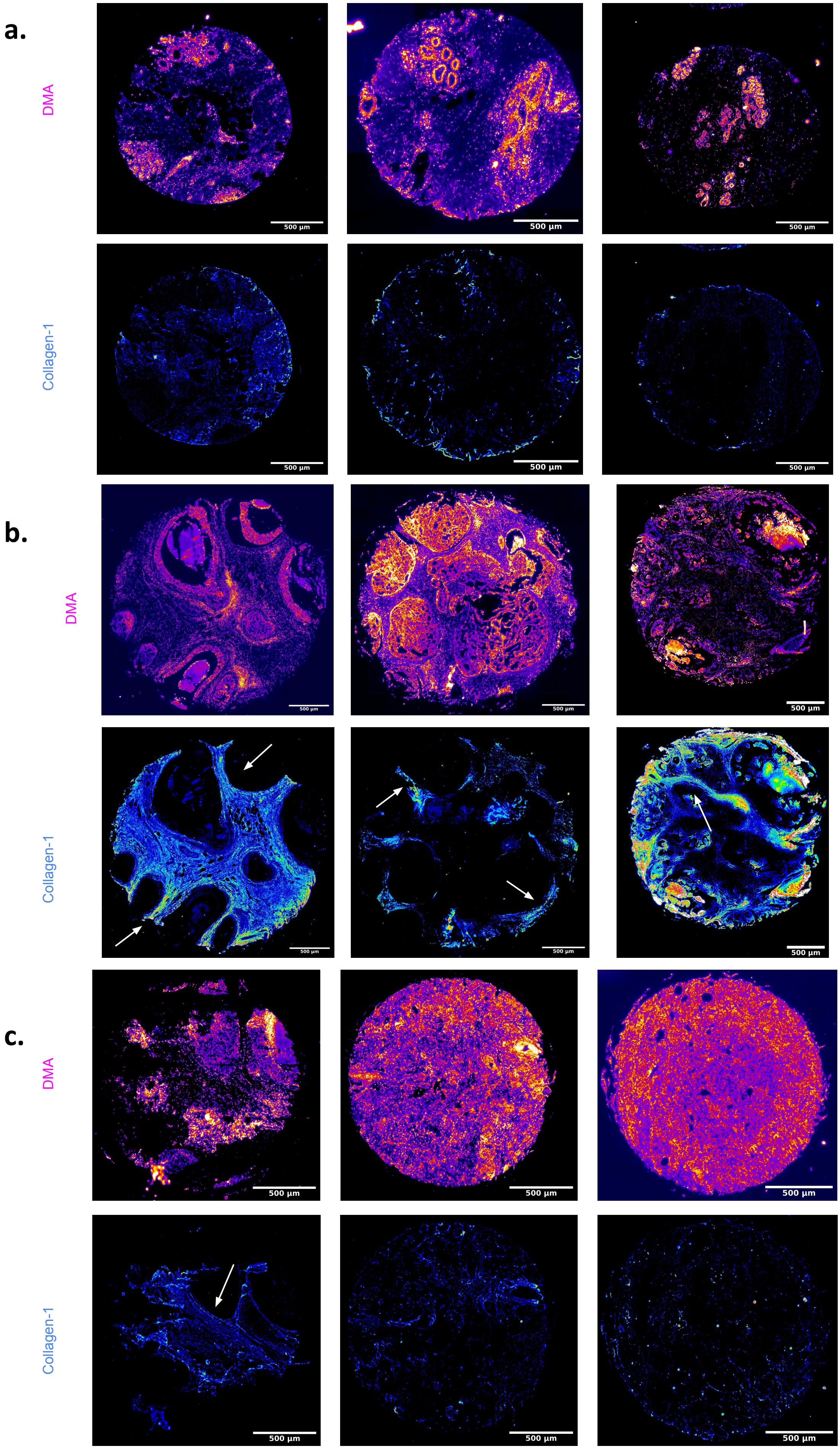

Figure S5D: Orientationally coupled regions are characteristic of early breast cancer states

Three representative images stained for DNA (top) and Collagen-1 (bottom) of TMA of breast biopsies clinically annotated as Normal (a), DCIS (b) and Invasive ductal carcinoma (c) . White arrows indicate regions of oriented collagen
